# Therapeutic efficacy and safety of artesunate + amodiaquine and artemether + lumefantrine in treating uncomplicated *Plasmodium falciparum* malaria in children on the rainy south-east coast of Madagascar[Fn FN1]

**DOI:** 10.1051/parasite/2023034

**Published:** 2023-08-30

**Authors:** Judickaëlle Irinantenaina, Gwénaëlle Carn, Dina Ny Aina Liantsoa Randriamiarinjatovo, Aina Nirina Harimanana, Seheno Razanatsiorimalala, Nicolas Ralemary, Maurice Randriarison, Celestin Razafinjato, Raphael Hotahiene, Milijaona Randrianarivelojosia

**Affiliations:** 1 Unité d’Epidémiologie et de Recherche Clinique, Institut Pasteur de Madagascar Antananarivo 101 Madagascar; 2 Drugs for Neglected Diseases initiative (DNDi) 1202 Geneva Switzerland; 3 Unité de Parasitologie, Institut Pasteur de Madagascar Antananarivo 101 Madagascar; 4 Direction Régionale de la Santé Publique Atsimo Atsinana Farafangana 309 Madagascar; 5 Service de Santé de District de Santé Publique Mananjary 317 Madagascar; 6 National Malaria Control Program, Ministry of Health Antananarivo 101 Madagascar; 7 Direction de Lutte contre les Maladies Transmissibles, Ministère de la santé publique Antananarivo 101 Madagascar; 8 Faculté des Sciences, Université de Toliara Toliara 601 Madagascar

**Keywords:** Artemether-lumefantrine, Artesunate-amodiaquine, Efficacy, Madagascar

## Abstract

Malaria is a major public health problem in Madagascar, particularly in coastal areas. We conducted a randomized, controlled, parallel-group study of artemisinin-based combination therapy (ACT) in Mananjary and Farafangana, two localities on the rainy south-east coast of Madagascar, from March to September 2018. The efficacy and safety of artesunate + amodiaquine (ASAQ) and artemether + lumefantrine (AL) were assessed according to the WHO protocol with a 28-day follow-up. Children aged 6 months to 14 years with uncomplicated *Plasmodium falciparum* malaria were randomized to receive ASAQ or AL for three days (1:1). 347/352 (98.5%) randomized patients reached the study endpoint on day 28. Crude adequate clinical and parasitological response (ACPR) rates were 100% (95% CI: 98.8–100%) in the ASAQ group and 96% (95% CI: 93.1–98.9%) in the AL group (per protocol population). However, the PCR-corrected ACPR rate was 97.7% (95% CI: 95.4–100%) in the AL group. Two cases of recrudescence and three of re-infection were observed. Mild and moderate adverse events, including gastrointestinal and/or nervous disorders, were reported in 11.9% (42/352) of patients. We found that ASAQ and AL were safe and efficacious for treating uncomplicated *P. falciparum* malaria. They may be used for treatment at health facilities and at the community level, and for mass drug administration campaigns.

## Abstract (fr)

Le paludisme demeure un problème majeur de santé publique à Madagascar notamment dans les régions côtières. Nous avons réalisé une étude multisite, randomisée, contrôlée, en groupes parallèles sur la combinaison thérapeutique à base des dérivés d’artémisinine (CTA) à Mananjary et Farafangana, deux localités sur la côte sud-est pluvieuse de Madagascar, de mars au septembre 2018. L’efficacité et la sécurité de l’artésunate + amodiaquine (ASAQ) et de l’artéméther + luméfantrine (AL) ont été évaluées selon le protocole de l’OMS avec un suivi de 28 jours. Des enfants âgés de 6 mois à 14 ans souffrant de paludisme non compliqué à *Plasmodium falciparum* ont été randomisés (1:1) pour recevoir ASAQ ou AL pendant trois jours. 347/352 (98,5 %) des patients randomisés ont pu être suivis jusqu’au jour 28. Le taux de réponse clinique et parasitologique adéquate (RCPA) était de 100 % (95 % CI : 98,8 – 100 %) dans le bras thérapeutique ASAQ et de 96 % (95 % CI : 93,1 – 98,9 %) dans le bras thérapeutique AL (population per protocole). Cependant, après correction par PCR, le taux de RCPA était de 97,7 % (95 % CI : 95,4 – 100 %) dans le bras thérapeutique AL. Deux cas de recrudescence et trois cas de réinfections ont été observées. Des effets indésirables légers et modérés, notamment des troubles gastro-intestinaux et/ou nerveux, ont été rapportés chez 11,9 % (42/352) des patients. Nos résultats démontrent que l’ASAQ et l’AL sont sûrs et efficaces pour le traitement du paludisme non compliqué à *P. falciparum*. Ces deux CTA peuvent par conséquent être utilisés pour traiter le paludisme dans les centres de santé et au niveau communautaire, et aussi pendant les campagnes de traitement de masse.

## Introduction

Malaria remains a major public health problem on the island of Madagascar, where perennial transmission occurs in coastal areas and *Plasmodium falciparum* is the predominant species [10, 11]. Effective case management, consisting of early diagnosis and prompt treatment with antimalarial drugs, is one key component of malaria elimination. Since 2005, the national malaria policy has adopted artemisinin-based combination therapies (ACT) and has recommended the combinations artesunate + amodiaquine (ASAQ) and artemether + lumefantrine (AL) for treating uncomplicated malaria. Over the last decade, studies have shown the efficacy of these drugs against *P. falciparum* malaria [7, 19, 25]. However, the emergence of resistance to antimalarial drugs remains a threat to malaria control [2, 13, 14] and it is crucial to monitor the efficacy of these ACTs. The World Health Organization (WHO) recommends monitoring of antimalarial drug resistance. Thus, to update the Ministry of Public Health on the efficacy of antimalarial drugs, we carried out a therapeutic efficacy study to assess the efficacy of ASAQ and AL on the south-eastern coastal areas of Madagascar.

## Methods

### Ethical considerations

This study was conducted according to the Declaration of Helsinki, guidelines on Good Clinical Practice, and existing national legal and regulatory requirements. The study protocol was submitted and approved by the Madagascar Biomedical Research Ethics Committee (013/MSANP/CERBM) on February 26, 2018. All participants (parents/guardians and patients aged 7–14 years) received oral and written information concerning the study. Written informed consent was obtained from parents or guardians of all participants. If patients were unable to sign, a fingerprint was applied to the consent form. Children aged between 7 and 14 years old gave written assent to participate in the study. All signed informed consent forms will be kept for 15 years after the end of the study.

### Study design

This study was a multisite, randomized, controlled study to assess the efficacy and tolerability of artesunate + amodiaquine (ASAQ) and artemether + lumefantrine (AL) combinations for treating uncomplicated *P. falciparum* malaria in children, based on the standard WHO protocol [29].

### Study sites

The study was conducted at two sites with perennial malaria transmission on the south-eastern coastal area of Madagascar (Antsenavolo and Kianjavato in the health district of Mananjary, and Vohitromby in the health district of Farafangana). At these sites, the average annual temperature ranges from 15 °C to 32 °C, and the average rainfall ranges from 1200 to 2500 mm [4, 5].

### Patients

Patients were included between March and September 2018 at a basic health center at each site. The study included patients infected with *P. falciparum,* residing in the study area for the duration of the trial, and according to the following criteria: aged between 6 months and 14 years, presenting an axillary temperature ≥ 37.5 °C or suffering from fever within the last 24 h, weighing ≥ 5 kg, having brachial circumference > 110 mm, hemoglobinemia ≥ 8g/dL, *P. falciparum* mono-infection with parasite density from 1000 to 100,000 asexual forms per microliter of blood, and capable of receiving oral treatment. Both study sites are in districts with moderate malaria transmission, defined as 50–100 cases per 1000 population per year [18]. In 2016, malaria parasite prevalence by microscopy among children 6–59 months of age was 9.0% in the zones encompassing Kianjavato, Antsenavolo and Vohitromby [12]. Patients with any clinical signs of severe malaria [16, 30] or any serious concomitant disease, such as cardiovascular disease, were excluded. Pregnant or breast-feeding women were also excluded, and a urine pregnancy test was performed in all women of child-bearing age. Successive enrolments in this study or simultaneously in any other clinical trial were also prohibited.

### Randomization and treatment

Patients were randomized to one of two treatment groups, namely once daily ASAQ and twice daily AL. Randomization was stratified according to patient age using separate computer-generated randomization lists. Three age strata were used: 6–59 months, 5–9 years, and 10–14 years. The treatment information was placed in a sealed envelope. Each study participant was randomly assigned (1:1) to receive either ASAQ or AL.

Treatment dosages were determined according to patient body weight [18, 31]. Treatment duration was three days. For the ASAQ regimen, one dose was administered daily for three days with an interval of 24 h. For the AL regimen, there was an interval of 8 h between the first and second dose on day 0, and an interval of 12 h between the two daily doses on day 1 and day 2. Tablets were administered orally with a small amount of drinking mineral water in the presence of a member of the study staff; patients were advised to resume a normal diet as soon as possible. All patients were monitored for 30 min after administration in order to ensure that the drug was not lost after any vomiting episode. When vomiting occurred, the same dose was re-administered. If vomiting re-occurred, the subject was withdrawn from the study and provided with a replacement treatment. In case of therapeutic failure or several signs, the patients were referred to the health center staff, and as per the national malaria control policy, the alternative treatment used was artesunate injection [23]. Paracetamol was administered to all patients with fever at or above 37.5 °C.

### Efficacy and safety evaluation

The WHO protocol with a 28-day follow-up to assess the therapeutic efficacy of anti-malarial drugs was used [29]. Follow-up visits were systematically performed on days 1, 2, 3, 7, 14, 21, and 28 after enrolment (Table 1)*.* Each visit consisted of a physical examination, combining evaluation of clinical safety and measurement of vital signs. Axillary temperature was measured using an electronic thermometer. Blood pressure and pulse were measured after a 10-min rest in sitting position. Blood samples were collected for parasitology (parasite count and PCR). On day 0 and day 28, hemoglobin concentration levels were measured with Hemocue^®^ Hb201 + (Hemocue, Ängelholm, Sweden), from capillary fingertip blood.

**Table 1:** Follow-up schedule.

Study parameters	Reference days								Unplanned visit

Day 0	Day 1	Day 2	Day 3	Day 7 ± 2d	Day 14 ± 2d	Day 21 ± 2d	Day 28 ± 2d
mRDT test	x								
Clinical examination	x	x	x	x	x	x	x	x	
Hemoglobin	x							x	
Physical examination/vital signs	x	x	x	x	x	x	x	x	x
Parasitological examination	x	x	x	x	x	x	x	x	x
Preparation of filter paper blood spot samples for PCR analysis	x	x	x	x	x	x	x	x	x

Clinical efficacy was assessed by grading the pre-existing clinical signs and combining them with the temperature values. Parasitological efficacy was based on asexual parasitemia, although a gametocyte count was also systematically performed. The following clinical symptoms were assessed systematically at each visit and graded as absent, mild, moderate, severe, or very severe: perspiration, headache, chills, pain (specifying topography), jaundice, asthenia, dizziness, anorexia, skin fold, skin rash, and pruritus. The severity grading scales were adapted from guidelines provided by the WHO (Toxicity Grading Scale for Determining the Severity of Adverse Events) [8, 27, 32]. Vomiting and diarrhea were assessed. The primary endpoint was the rate of adequate parasitological and clinical response after PCR correction on day 28, following the 2009 WHO protocol [29]. Clinical safety was monitored through regular patient interviews about the occurrence of adverse events since the previous visit.

### Study withdrawal

Patients, parents, or guardians were free to withdraw from the study or to stop taking treatment at any moment, irrespective of the reason. Patients were withdrawn following the investigator’s decision in case of onset of any danger signs of severe malaria or any adverse event justifying treatment discontinuation. Early study withdrawal was documented by the investigator on the case report form (CRF). When possible, patients were evaluated according to the end-of-study visit procedures. Patients presenting severe disease or adverse events were referred for medical care.

The study team made a phone call to remind the patient prior to each visit. At each visit, patients were given an allowance for travel expenses. In cases where a patient missed a visit, the study team visited the patient’s home up to three times. If the patient was not available for the follow-up after three attempts, they were declared “lost to follow-up”. For patients lost to follow-up, the CRF was completed up to the last visit attended.

No patients prematurely withdrawn from the study were replaced.

### Laboratory analysis

At enrolment and at successive follow-up visits, thin and thick smears were obtained and stained with May Grünwald-Giemsa 10%. They were examined at the sites by dual reading to identify *P. falciparum* mono-infection and determine parasite density. Finger prick blood samples were collected on filter paper (DBS Whatman^®^ 903 protein saver cards, batch No.: 7088517W162, USA).

Microscopy: parasite densities were calculated by counting the number of asexual and sexual *P. falciparum* parasites after observing 500 leukocytes and then converting this figure into parasites (trophozoites and/or gametocytes) per microliter of blood, assuming an average leukocyte count of 8000/μL.

For quality control purposes, all slides obtained at enrolment and at successive follow-up visits for Mananjary and Farafangana were analyzed by microscopy practitioners at IPM using a procedure that was blinded to the original result. Microscopy was considered negative if no parasites were seen after observing 1000 leukocytes.

#### PCR analysis

DNA extraction was performed from DBS using QIAGEN kits (QIAGEN Inc., Hilden, Germany), according to the manufacturer’s instructions. The analyses were performed at the Parasitology Unit of the Institut Pasteur de Madagascar. For participants with recurrent parasitemia after day 7, paired polymerase chain reaction (PCR) blots (from day 0 and the day of parasitemia recurrence) were analyzed for parasite merozoite surface proteins (MSP-1 and MSP-2) and glutamate rich protein (GLURP) to distinguish between reinfection and recrudescence, as described elsewhere [20, 24]. Day 0 and failure day alleles of the *msp1*, *msp2*, and *glurp* gene loci were compared. The PCR was performed using the following primer pairs MSP1: MSP1C1 = 5′–GTACGTCTAATTCATTTGCACG–3′ and MSP1D1 = 5′–CACATGAAAGTTATCAAGAACTTGTC–3′ for PCR1, MSP1C2 = 5′–GATTGAAAGGTATTTGAC–3′ and MSP1D2 = 5′–GCAGTATTGACAGTTTATGG–3′ for PCR2; MSP2 = MSP2D1: 5′–GAAGGTAATTAAAACATTGTC–3′ and MSP2C1 = 5′–GAGGGATGTTGCTGCCAACAG–3′ for PCR1, MSP2D2 = 5′–GAGTATAAGGAGAAGTATG–3′ and MSP2C2 = 5′–CTAGAACCATGCATATGTCC–3′ for PCR2; and GLURP: GlurpC1 = 5′–ACATGCAAGTGTTGATCC–3′ and GlurpC2 = 5′–GATGGTTTGGGAGTAACG–3′ for PCR1 and GlurpG1 = 5′–TGAATTCGAAGATGTTCACACTGAAC–3′ and GlurpG3 = 5′–TGTAGGTACCACGGGTTCTTGTGG–3′ for PCR2. Three microliters of DNA were amplified for 35 cycles during the primary PCR. Two microliters of the product of that amplification were used for another 35 cycles of PCR. Each PCR was performed with 1 μM of each primer, 200 μM of each dNTP, and 2.5 mM MgCl2. The sizes of the amplification products were determined by agarose gel electrophoresis using Quantity One^®^ software v. 6.4.1 (Bio-Rad Laboratories, Hercules, CA, USA). Possible outcomes were i) recrudescence, if the alleles of the pre-treatment and post-treatment samples were the same for *msp1*, *msp2*, and *glurp*; ii) reinfection, if the alleles of the pre-treatment and post-treatment samples were distinct for any of these three loci; iii) mixed recrudescence and reinfection, if similar alleles were found in the pre-treatment and post-treatment samples for all the markers as mentioned above, but with additional distinct alleles identified; and iv) indeterminate, if either or both the pre-treatment and post-treatment samples could not be amplified. Mixed recrudescent and re-infection cases were computed as recrudescent [35].

### Sample size calculation

The number of subjects was calculated based on 28-day PCR corrected efficacy. We took into account the success rate hypothesis (95%), a confidence interval of 95%, a type I (α) error of 5% and a type II (β) error of 20%, and a loss to follow-up rate of 15%. A minimum of 86 patients per arm was needed for each study site.

### Statistical analysis

Data were recorded using the WHO-standard template using the ODK (Open Data Kit) system on an Android tablet and added to the database in real time. Data cleaning was done regularly to identify and fix any data that were incorrect, inaccurate, or incomplete. Statistical analysis was performed using RStudio version 4.2.2 (The R Foundation for Statistical Computing).

The primary endpoint was 28-day PCR-corrected treatment efficacy. Treatment outcomes were assessed according to the WHO guidelines [29] for early treatment failures (ETF; danger signs, complicated malaria, or failure to adequately respond to therapy on days 0–3), late clinical failures (LCF; danger signs, complicated malaria, or fever and parasitemia on days 4–28 without previously meeting criteria for ETF), late parasitological failure (asymptomatic parasitemia on days 4–28 without previously meeting criteria for ETF or LCF), and adequate clinical and parasitological response (ACPR; absence of parasitemia on day 28 without previously meeting criteria for ETF, LCF, or LPF) [29]. Secondary endpoints included parasitemia and gametocyte carriage, and hemoglobin level.

Descriptive analysis was conducted using frequency/percentage for qualitative variables and median/interquartile interval (IIQ) for quantitative variables. Categorical variables were compared with the *χ* ^2^ test or Fisher’s exact test, and quantitative variables with the Wilcoxon test. The uncorrected and PCR-corrected per protocol efficacy for each site and drug was calculated by dividing the number of participants classified as ACPR by all participants reaching a study outcome. The sum of posterior probabilities of recrudescence was used to calculate the total number of recrudescent infections for the PCR corrected analysis.

The PCR-corrected per-protocol population consisted of all patients without a major protocol deviation, who had a primary endpoint at day 28, who did not have any reinfection. For Kaplan–Meier cumulative efficacy estimates, re-infections, lost to follow-up, and patients who withdrew were censored on the day of re-infection or the last day of follow-up. Posterior sampling was used to generate the PCR-corrected Kaplan–Meier estimates and 95% confidence intervals using the posterior probabilities of recrudescence.

## Results

**Figure 1:**
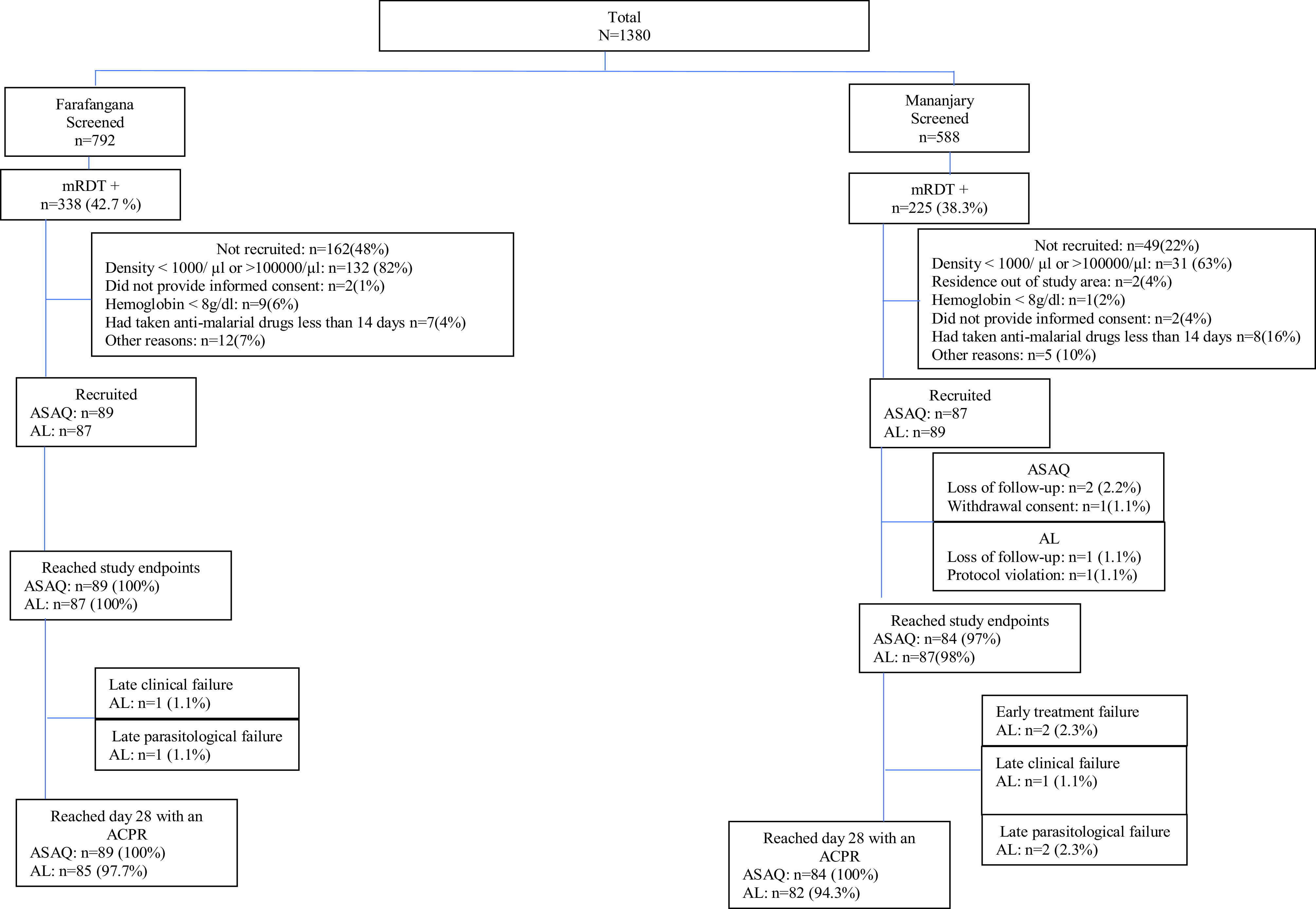
Participant information, antimalarial therapeutic efficacy, Madagascar 2018.

During the study period, 352 of 1380 eligible children were randomized for inclusion in one of the two therapeutic arms, 176 in the ASAQ arm and 176 in the AL arm. Overall, 50.6% (89/176) of patients in the ASAQ group and 50% (88/176) in the AL group were female. Table 2 displays patient features at enrolment. Five patients (1.4%) did not complete visits up to day 28, three due to loss to follow-up, one due to protocol deviation, and one patient withdrew (Table 3). On day 28, the per-protocol population consisted of 347 patients (50.7% female and 49.3% male). The flow of patients through the study is displayed in Figure 1

**Table 2:** Characteristics of participants at enrollment (day 0).

	Farafangana		Mananjary	

ASAQ, *n* = 89	AL, *n* = 87	*p*-value	ASAQ, *n* = 87	AL, *n* = 89	*p*-value
Age (months), median (IIQ)	84 (53–108)	72 (48–108)	0.9	96 (50–132)	83 (53–132)	>0.9
Weight (kg), median (IIQ)	17 (13–21)	17 (13–21)	0.7	19 (14–27.5)	18.5 (14–28)	0.9
Female, *n* (%)	45 (50.6)	46 (52.9)	0.8	44 (50.6)	42 (47.2)	0.7
Parasitemia (parasites/μL), median (IIQ)	16,945 (7,392–38,543)	24,404 (8,194–37,077)	0.6	12,972 (4,836–40,034)	19,156 (8,787–31,725)	0.3
Temperature (°C), median (IIQ)	37.6 (36.9–38.7)	37.6 (36.8–38.7)	0.8	37.8 (36.8–38.5)	37.9 (36.9–38.6)	0.5
Gametocytemia, *n* (%)	9 (10.1)	8 (9.2)	0.8	2 (2.2)	6 (6.7)	0.3
Gametocyte carriers, median (IIQ)	48 (32–86)	75.5 (34–240)	0.7	47.5 (39.8–55.3)	39 (27–63)	0.6
Hemoglobin (g/dL), median (IIQ)	10 (9.2–11.3)	10.2 (9–11.2)	0.8	11.7 (10.3–13)	11.7 (10.1–13.2)	0.7

AL: artemether-lumefantrine, ASAQ: artesunate-amodiaquine.

**Table 3:** Characteristic of participants enrolled and completing follow-up, Madagascar 2018.

	Farafangana	Mananjary

ASAQ, *n* = 89	AL, *n* = 87	ASAQ, *n* = 84	AL, *n* = 87
Crude results				
Early treatment failure, *n* (%)	0	0	0	2 (2.2)
Late clinical failure, *n* (%)	0	1 (1.2%)	0	1 (1.1)
Late parasitological failure, *n* (%)	0	1 (1.2%)	0	2 (2.2)
RCPA	89 (100%)	85 (97.7%)	84 (100%)	82 (94.3%)
Corrected results				
Early treatment failure, *n*	0	0	0	2
Recrudescence, *n*	0	1	0	1
Day 21	0	1	0	1
Day 28	0	0	0	0
Reinfection, *n*	0	1	0	2
Day 21	0	0	0	0
Day 28	0	1	0	2
RCPA	89 (100%)	85 (98.8%)	84 (100%)	82 (96.5%)
Hemoglobin day 0 (g/dL), median (IIQ)	10 (9.2–11.3)	10.2 (9–11.2)	11.7 (10.3–13)	11.7 (10.1–13.2)
Hemoglobin day 28 (g/dL), median (IIQ)	10.6 (8.2–11.6)	11.6 (10.6–12.6)	12.2 (11.3–13.3)	12.4 (11.2–13.6)

.

### Clinical and parasitological responses to treatments

PCR-corrected and uncorrected clinical and parasitological cure rates on day 28 using a Kaplan–Meier estimate of survival curve are shown in Table 5. The crude cumulate efficacy in the AL arm was 97.7% (95%CI: 94.6–100) in Farafangana and 94.3% (95%CI: 89.5–99.3) in Mananjary. By gender, in the AL arm, crude efficacy was 95.7% (95%CI: 89.9–100) in the female group and 100% in the male group in Farafangana, and 97.6% (95%CI: 93.1–100) and 91.1% (95%CI: 83.2–99.8), respectively in Mananjary. At day 1, two cases of early treatment failure were identified. Five (3 female and 2 male) cases of late failure were identified: 1 at day 21 and 4 at day 28. Three (2 males and 1 female) of the late treatment failures were reinfections at day 28 and two female patients was classified as recrudescence at day 21 and day 28. Both recrudescence cases were in the under-five years age group. After exclusion of reinfections, the corrected 28-day efficacy of AL was 96.5% (95% CI: 92.6–100) in Mananjary and 98.8% (95% CI: 96.6–100) in Farafangana. Overall, the efficacy of AL was estimated at 97.7% (95%CI: 95.4–100). There were no significant differences in age or gender between the two group (*χ*^2^ test, *p* = 0.1).

**Table 5:** Efficacy of first-line antimalarial drugs at day 28, Madagascar 2018.

	Farafangana	Mananjary

ASAQ	AL	ASAQ	AL

%	95%CI	%	95%CI	%	95%CI	%	95%CI
Uncorrected PCR								
Per protocol	100	95.9–100	94.3	89.5–99.3	100	95.7–100	94.3	89.5–99.3
Kaplan–Meier	100	95.9–100	94.3	89.5–99.3	100	95.8–100	94.4	89.6–99.3
Corrected								
Per protocol	100	95.9–100	98.8	96.6–100	100	95.7–100	96.5	92.6–100
Kaplan–Meier	100	95.9–100	98.8	96.6–100	100	95.8–100	96.6	92.9–100

**Table 6:** Efficacy of first-line antimalarial drugs at day 28 stratified by site, age and study arm, Madagascar 2018.

	Study arm							
Per protocol				Kaplan–Meier			
ASAQ		AL		ASAQ		AL	
%	95%CI	%	95%CI	%	95%IC	%	95%IC
Region								
Antsenavolo	100	87.2–100	100	86.7–100	100	87.2–100	100	87.2–100
Kianjavato	100	93.7–100	94.9	89.5–100	100	94–100	95.1	89.9–100
Vohitromby	100	95.9–100	98.8	96.6–100	100	95.9–100	98.8	86.6–100
Age (years old)								
0.5–4	100	93.2–100	96.2	91.2–100	100	93.2–100	96.2	91.2–100
5–14	100	63–100	98.3	96–100	100	63–100	98.4	96.1–100
Gender								
Female	100	95.9–100	97.7	94.6–100	100	95.9–100	97.7	94.7–100
Male	100	95.7–100	97.6	94.4–100	100	95.7–100	97.7	94.7–100

In the ASAQ arm, the uncorrected cumulate efficacy was 100% (95%CI: 95.9–100) in Mananjary and Farafangana. Overall, the efficacy of ASAQ was estimated at 100% (95%CI: 97.8–100). There were no significant differences in therapeutic response rates adjusted by region and age (Table 6)*.*

### Parasitic clearance and resolution of clinical symptoms

**Figure 2:**
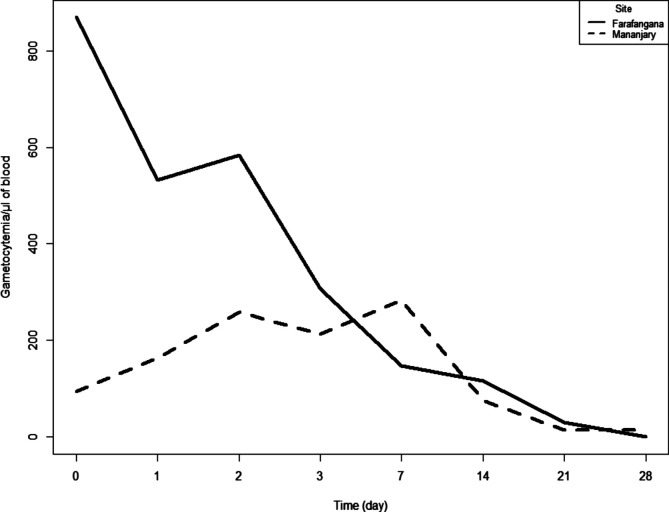
Gametocyte clearance in the ASAQ arm at both study sites, antimalarial therapeutic efficacy, Madagascar 2018.

**Figure 3:**
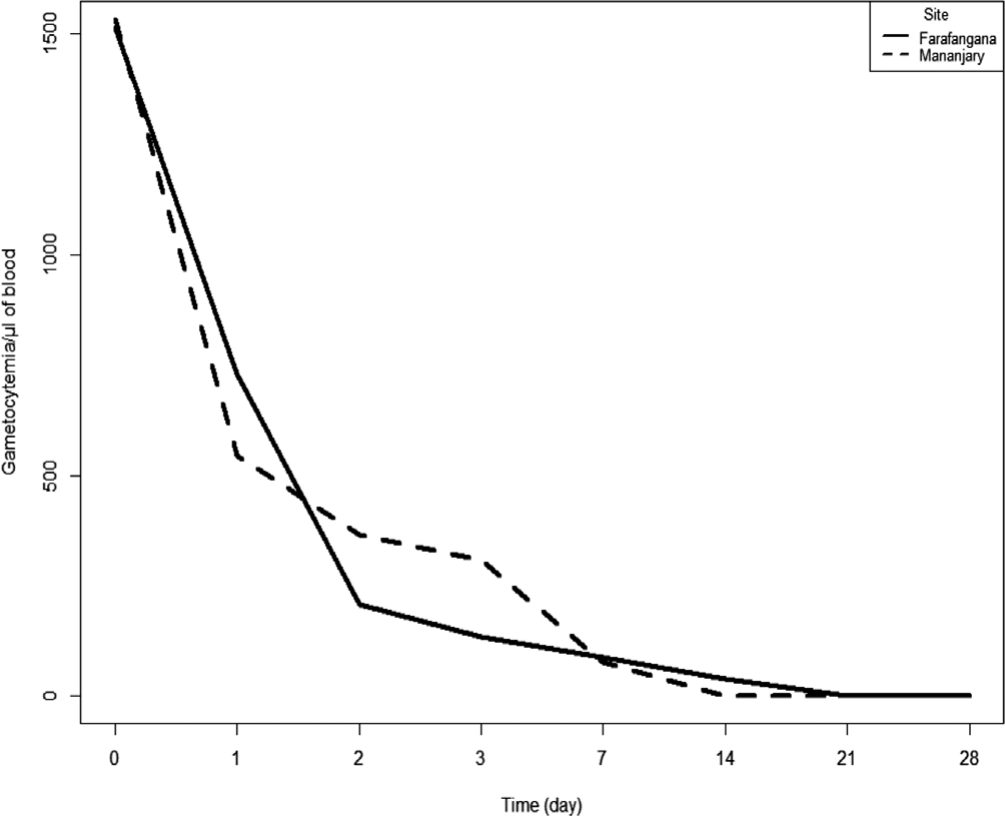
Gametocyte clearance in the AL arm at both study sites, antimalarial therapeutic efficacy, Madagascar 2018.

At enrolment, in the AL arm the median (IIQ) parasite densities varied from 19,156 parasites/μL of blood (8787–31,725) in Mananjary to 24,404 parasites/μL of blood (8193–37,076) in Farafangana, and in the ASAQ arm the median (IIQ) parasite densities varied from 12,972 parasites/μL of blood (4835–40,033) in Mananjary to 16,945 parasites/μL of blood (7392–38,543) in Farafangana. Ten patients remained parasitemic on day 3: 0.6% (1/176) in the ASAQ arm and 5.1% (9/176) in the AL arm (Table 4). The gametocyte density decreased from day 1 and reached zero on day 28 for most patients in the ASAQ arm in Farafangana, and remained present but at low density for a few patients in Mananjary (Fig. 2). In the AL arm, the gametocyte density decreased and reached zero on day 14 in Farafangana and on day 21 in Mananjary (Fig. 3).

**Table 4:** Parasite clearance, Madagascar 2018.

	Farafangana		Mananjary	

ASAQ, *n* = 89	AL, *n* = 87	*p*-value	ASAQ, *n* = 87	AL, *n* = 89	*p*-value
Day 1						**0.007^1^**
*n* (%)	39 (43.8)	50 (57.4)	0.07	62 (71.3)	74 (83.1)	0.03
Density (median [IIQ])	300 (205–1420)	468.5 (245.2–1256.5)	0.5	250.5 (87–600.8)	524.5 (179.2–3025.8)	< 0.001
Day 2						**0.003^1^**
*n* (%)	3 (3.3)	10 (11.5)	0.04	19 (21.8)	33 (37.1)	0.02
Density (median [IIQ])	261 (182–678)	67 (25.7–123.5)	0.08	82 (43–140.5)	75 (39–160)	>0.9
Day 3						**0.010^1^**
*n* (%)	0	1 (1.1)	0.5	1(1.1)	8 (9)	0.03
Density (median [IIQ])	0	63 (–)	0.5	11 (–)	35.5 (25.5–43.5)	0.02

1 Overall *p*-value parasite clearance between the 2 treatment groups.

### Drug safety

Overall, 11.9% (42/352) patients who reached a study endpoint presented at least one emergent adverse event. Among these adverse events, 54.8% (23/42) were in the ASAQ arm and 45.2% (19/42) in the AL arm. In the ASAQ arm, the most frequently reported AEs included: cough 30.4% (7/23), abdominal pain 21.7% (5/23), vomiting 17.3% (4/23), headache 13.04% (3/23), edema 4.3% (1/23), sleep disorder 4.3% (1/23), eye trauma 4.3% (1/23) and anorexia 4.3% (1/23). In the AL arm, the most frequently reported AEs included: cough 31.5% (6/19), flu-like symptoms 21.05% (4/19), abdominal pain 10.5% (2/19), fever (10.5% (2/19), trauma 10.5% (2/19), vomiting 5.2% (1/19), headache 5.2% (1/19), adenopathy 5.2% (1/19), and anorexia 5.2% (1/19). Of all adverse events, two in the AL arm fulfilled the severity criteria. None of the adverse events were considered treatment-related by the investigators. No significant difference was observed between the two treatment groups in terms of presence of adverse events (*χ* ^2^ test, *p* = 0.3). Table 7 below summarizes the clinical characteristics of the patients included in the study between day 0 and day 7.

**Table 7:** Clinical characteristics from day 0 to day 7, Madagascar 2018.

	ASAQ	AL

Day 0	Day 1	Day 2	Day 3	Day 7	Day 0	Day 1	Day 2	Day 3	Day 7
Fever										
ATV	27	1	–	1	–	27	3	–	–	–
KVT	60	2	1	–	1	62	12	–	–	1
VHT	89	4	–	–	–	87	5			
Abdominal pain										
ATV	4	–	1	2	–	4	2	–	–	1
KVT	8	4	1	–	–	14	2	2	–	–
VHT	52	12	1	1	1	48	4	1	–	–
Vomiting										
ATV	13	–	–	–	1	12	–	–	–	–
KVT	29	3	–	1	–	20	1	–	–	–
VHT	21	–	–	–	1	18	–	–	–	1
Cough										
ATV	3	3	2	3	3	4	2	2	–	–
KVT	8	5	3	2	4	10	2	3	2	1
VHT	31	19	15	8	3	41	22	11	6	3
Anorexia										
ATV	5	2	–	–	–	5	1	–	–	–
KVT	14	3	1	–	1	20	4	2	–	–
VHT	33	6	–	–	–	26	3	–	–	–
Headache										
ATV	12	1	1	–	–	9	1	–	–	1
KVT	34	7	1	–	–	42	10	1	–	–
VHT	66	6	–	–	1	52	5	1	1	2
Rhinorrhea										
ATV	1	2	1	1	1	2	1	1	–	–
KVT	4	2	1	1	3	7	1	1	1	–
VHT	–	–	–	–	–	1	–	–	–	–
Nausea										
ATV	2	–	–	–	–	1	–	–	–	–
KVT	5	1	–	–	–	9	1	–	–	–
VHT	1	–	–	–	–	3	–	–	–	–
Asthenia										
ATV	–	–	–	–	–	2	–	–	–	–
KVT	6	–	–	–	–	2	27	1	–	–
VHT	43	27	9	3	–	43	22	6	–	–
Diarrhea										
ATV	–	–	–	–	–	–	–	–	–	–
KVT	1	2	2	–	–	3	–	1	–	–
VHT	–	–	–	–	–	3	–	–	–	–
Dizziness										
ATV	–	–	–	–	–	1	–	–	–	–
KVT	3	1	–	–	–	2	1	–	–	–
VHT	6	–	–	–	–	7	–	–	–	–

ATV: Antsenavolo/Mananjary, KVT: Kianjavato/Mananjary, VHT: Vohitromby/Farafangana.

Data for hemoglobin values at day 0 and day 28 were available for 344 individuals; no statistical difference was observed between treatment arms at day 0 (*p* = 0.1) and day 28 (*p* = 0.5). An increase in hemoglobin values was observed for 217 individuals (110 individuals in the ASAQ arm and 107 individuals in the AL arm), a decrease for 85 individuals (41 and 44, respectively), and no change for 42 individuals (22 and 20, respectively). The means of hemoglobin level differences between day 0 and day 28 were statistically different between the 2 treatment arms (*t* test, *p* < 0.001).

## Discussion

Progress towards the elimination of malaria in Madagascar depends on the involvement of the health system in rapid and effective case management. This requires not only the rapid diagnosis of suspected cases, but also the effectiveness of antimalarial drugs administered in each confirmed case. The main objective in this multisite, randomized, controlled, parallel-group trial in children with uncomplicated *P. falciparum* malaria conducted in 2018, was to assess the efficacy of ASAQ and AL after 28 days of follow-up, in accordance with the minimum standard duration recommended by the 2009 WHO protocol [29], and to limit the risk of loss to follow-up related to moving around, knowing that the patients had to walk to reach the health center.

**Figure 4:**
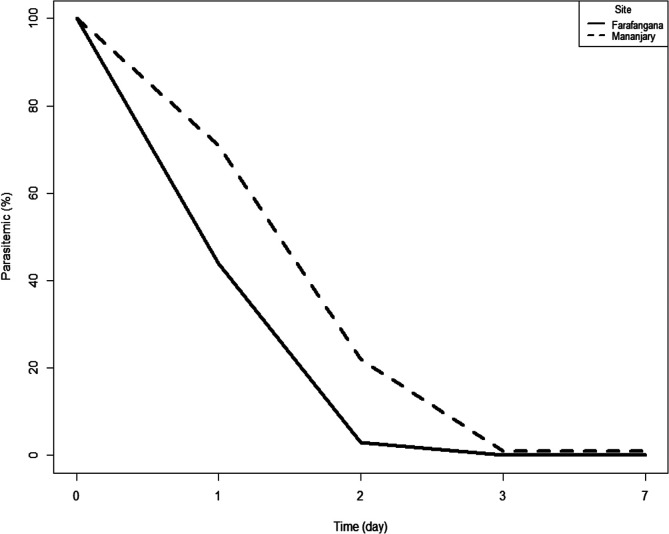
Parasite clearance in the ASAQ arm at both study sites, antimalarial therapeutic efficacy, Madagascar 2018.

**Figure 5:**
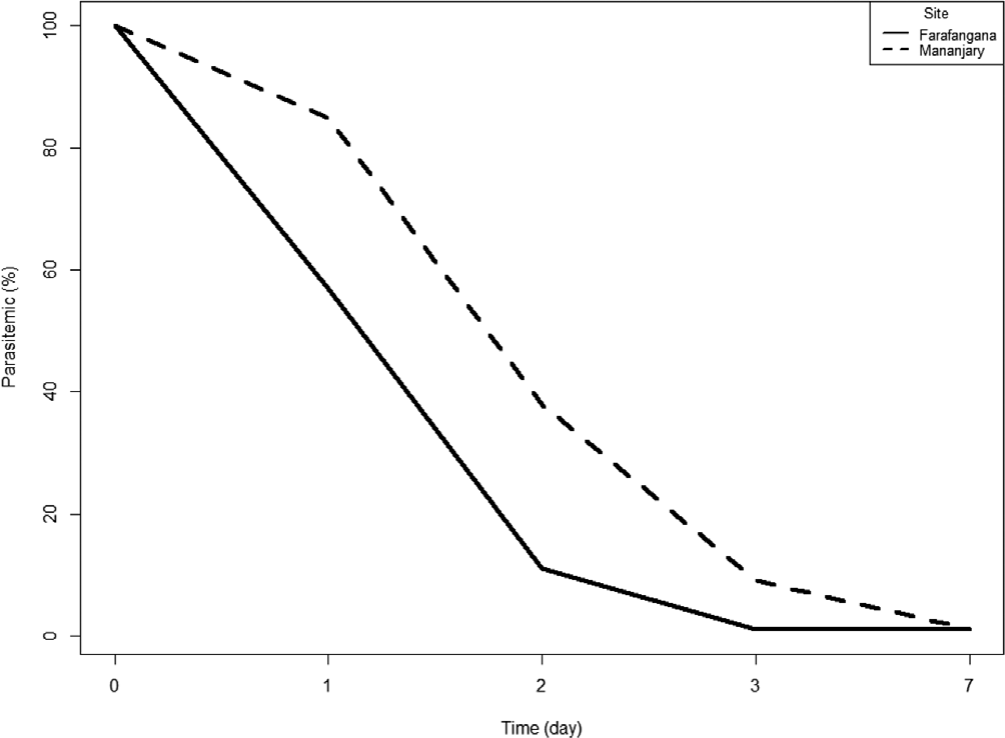
Parasite clearance in the AL arm at both study sites, antimalarial therapeutic efficacy, Madagascar 2018.

The treatment responses reported in this study demonstrate the good efficacy of ASAQ and AL. They are consistent with those of previous studies conducted in Madagascar [7, 25, 35]. Both ASAQ and AL exceeded the 90% threshold at which WHO recommends considering a change in first line anti-malarial drugs [29]. Consistent with the aforementioned studies, we found that ASAQ induced rapid parasitic clearance within three days and a more than 50% decrease of gametocytemia within two weeks, reaching complete elimination of gametocytes in most patients at the end of the study (day 28). However, parasitemia resolving in both groups was 81% at day 2 and 97% at day 3, and the complete disappearance of parasites was observed at day 7. This is consistent with a study from 2012 to 2013 in Niamtougou in Togo [9]. The proportion of children remaining parasitemic on day 3 was statistically significantly higher in the AL group (*χ*^2^ test, *p* = 0.01) than in the ASAQ group (Figs. 4 and 5). Complete parasite clearance in the AL arm was later than in other similar studies [1, 6, 20]. However, the emergence and spread of *P. falciparum* resistance to artemisinin in Asia is alarming [2, 13]. We should also bear in mind that human mobility or *in situ* selection may explain the emergence of drug-resistant malaria parasites. The spread of the *P. falciparum pfcrt* mutant from southeast Asia to Africa has already been demonstrated [17]. Also, the recent identification of parasites harboring a *pfk13* mutation, R561H, with associated delayed clearance after treatment with AL in Rwanda, underscores the importance of continued monitoring for *pfk13* mutations in African TESs [26, 28]. No studies with gender-related analysis of antimalarial efficacy were found, but one study in rats showed that sex did not significantly affect the antimalarial activity of treatments [33]. In contrast, in a study carried out in Malawi in 2014, higher efficacy was shown in the female group [21]. The observed cases of reinfection in this study, despite their low proportion, indicate perennial transmission of malaria parasites in these areas.

Our results demonstrated that both assessed treatment regimens (ASAQ and AL) were well tolerated. The events observed during the assessment were expected and have already been described with this type of antimalarial. Abdominal pain, vomiting, headache and anorexia were the symptoms frequently reported by patients who took the artemisinin derivative in both groups. The tolerability of the treatment regimens was similar in our study. In contrast, in Cote d’Ivoire for example, it was shown that AL is more tolerable than ASAQ [15, 34].

At both sites, the uncorrected and corrected efficacy was lower in the AL group than in the ASAQ group. It is known that AL administration unaccompanied by fatty foods, as recommended by the manufacturer, may account for this difference that has been found in various studies, because both the effectiveness and tolerability of AL are better when the drug is taken with higher fat foods [3, 22].

## Conclusion

Our results demonstrate that the fixed-dose combinations ASAQ and AL are safe and efficacious for treating uncomplicated *P. falciparum* malaria in the south-eastern parts of Madagascar. We believe that ASAQ and AL may be used as first-line treatments to support the national policy plan to strengthen malaria control, with the aim of eliminating this disease in Madagascar. Also, ASAQ and AL could be used for mass drug administration to save lives during the frequent malaria outbreaks on the eastern coast of Madagascar.
